# The Early Diagnosis of Pulmonary Tuberculosis from the View Point of General Practice

**Published:** 1915-05-15

**Authors:** H. Hyslop Thomson

**Affiliations:** County Tuberculosis Officer, Hertford, formerly Medical Superintendent Liverpool Sanatorium; Author of "Consumption in General Practice," &c.


### THE EARLY DIAGNOSIS OF PULMONARY TUBERCULOSIS.

### From the View Point of General Practice.

By H. HYSLOP THOMSON, M.D., D.P.H., County Tuberculosis Officer, Hertford, formerly Medical Superintendent Liverpool Sanatorium; Author of "Consumption in General Practice," &c.

The whole problem of the control and successful treatment of pulmonary tuberculosis rests upon the detection of the disease in its early stages before the resistance and recuperative powers of the tissues have become seriously impaired by the establishment of vicious circles. There are two main channels by which tubercle bacilli reach the lung—one through the air passages by inhalation, and the other through the tonsils and cervical lymphatic glands to the pleura, or by the bronchial glands to the root of the lung. In the lung itself the tubercle bacillus may find a resting-place in one of four sites—namely, the sub-pleural lymphatic tissue, the peri-bronchial tissue, the perivascular tissue, or the walls of the lung alveoli. Many of the signs and symptoms of early pulmonary tuberculosis can be referred to the presence of tuberculous deposits in one or other of these sites. Early pulmonary tuberculosis may be closed or open, latent or active. It is, therefore, possible to speak of anatomical pulmonary tuberculosis as opposed to clinical tuberculosis. In the former type no morbid indications present themselves, but in the latter type certain signs and symptoms are present.

### The Pre-tuberculous Stage.

A pre-tuberculous stage has frequently been referred to in the development of pulmonary tuberculosis. This term can only correctly be applied to the period of incubation of the tubercle bacillus, or to that phase of diminished resistance which invariably precedes the development of the bacillus in the human body. It is a useful aid to diagnosis to search for evidence of impaired resistance to the development of the tuberculous process. A family history of tuberculosis points to the possible inheritance of a diminished resistance to the disease. Certain morbid conditions are particularly liable to leave the portals open for the tubercle bacillus. Acute respiratory diseases, influenza, enteric fever, insanity, neurasthenia, diabetes, and alcoholism are all liable to pave the way for infection with tubercle.

### Exposure to Infection.

A history of exposure to infection frequently directs the thoughts of the physician to the possibility of tuberculosis. The incidence of pulmonary tuberculosis is higher in contacts than in non-contacts. Moreover, patients themselves frequently seek medical advice because they have been in prolonged or intimate contact with those suffering from the disease. The possibility of auto-infection as a cause of pulmonary tuberculosis must also be borne in mind. The evidence of a past or present non-pulmonary tuberculosis should suggest the law of Lewis, which states that, given a non-pulmonary tuberculous lesion, the next structure to be involved will, in the majority of cases, be the pulmonary tissue.

### Stigmata of Tuberculous Infection.

The stigmata of tuberculous infection which are of value as an aid to diagnosis include the following: (1) palpable lymphatic glands in the retro-pharyngeal and supra-clavicular regions; (2) enlarged veins radiating over one or both sides of the chest in front, and a festoon of dilated veins in the cervico-dorsal region behind; (3) long eyelashes and excessive growth of fine hair on the back; (4) conformity to the well-known phthisical, scrofulous or neurasthenic type; (5) unilateral flushing of the face and inequality of pupils. In the routine examination of contacts and suspects amongst children the presence and combination of certain of these stigmata will be found of the greatest value in arriving at a correct diagnosis.

### Localising Indications of Early Pulmonary Tuberculosis.

Of the classical symptoms of early pulmonary tuberculosis cough is by far the most frequent. Patients with incipient pulmonary tuberculosis frequently state that they have no cough. This is usually due to the fact that the cough is so slight or infrequent that it is overlooked. Suspected patients should be questioned on the following points: (1) as to the presence of coughing or hawking on rising in the early morning; (2) as to whether a change of temperature induces coughing; and (3) as to whether coughing is ever produced by violent muscular or respiratory movements. When cough is present it may or may not be accompanied by expectoration. This is usually scanty and mucoid in character, and tubercle bacilli are not detected in the majority of cases. In a certain type of case expectoration is absent because it is swallowed. Young children, insane people, and nervous or hysterical females, when they develop pulmonary tuberculosis, persistently swallow their sputum. Hæmoptysis not infrequently occurs as an early symptom and invariably leads the patient to seek medical advice. It may occur in apparently healthy individuals, and be the first overt symptom of the disease. Frequently, however, the spitting of blood is due to extra-pulmonary causes, or it may result from some morbid condition of the lungs other than tuberculosis. Dyspnœa, when it occurs as a symptom in early pulmonary tuberculosis, is invariably associated with acute onset and progressive activity. Pain is frequently present. In many cases it is associated with fibrosis and contraction, and it is therefore not an unfavourable sign. The pain of pleuritis and that due to the rupture of pleural adhesions is sharp and distinctive. Hyperæsthesia may be present, but it is of little help in diagnosis. It is frequently observed in neurotic and hysterical patients who imagine they are victims of tuberculosis.

### Evidence of Constitutional Disturbance.

The constitutional symptoms met with in early pulmonary tuberculosis are due to the presence in the systemic circulation of the toxic products of the tubercle bacillus, and their character and degree of severity will be proportional to the character and amount of toxin present. Temperature variations occur early in the course of the disease. The morning temperature in health ranges from 97 deg. Fahr. to 97.6 deg. Fahr., and the evening temperature from 97.8 deg. to 98.4 deg., the amplitude rarely exceeding one degree. In early pulmonary tuberculosis there occur various deviations from this normal standard. A persistent sub-normal temperature with an increased amplitude is not infrequently met with. More usually, however, the amplitude is increased by a rise in the early afternoon or evening temperature. At the same time the patient becomes susceptible to auto-inoculation, so that increased muscular effort is followed by a rise in temperature. The pulse-rate may be quickened early in the course of the disease, but its chief value at this stage is in relation to prognosis. Headache intensified by muscular effort, persistent languor, and muscular wasting are significant early symptoms. Anæmia —especially in a young male—and dyspeptic symptoms are important indications of early tuberculosis. The dyspeptic symptoms may be so marked as to suggest the presence of gastric disease, and lead the observer to a wrong conclusion as to the seat of trouble. Neurasthenic symptoms are frequently due to the toxin of the tubercle bacillus, but they are not always associated with well-marked physical signs.

### Early Physical Signs.

Cases of early pulmonary tuberculosis may be divided into two classes: (*a*) those in which adventitious sounds are present; and (*b*) those in which adventitious sounds are absent. Deviation from the normal in the character of the breath-sounds is more frequently detected than the presence of adventitious sounds. Moreover, it occurs earlier in the course of the disease. In certain cases, however, adventitious sounds may be detected at an early stage. When the disease originates in the walls of the lung alveoli a series of fine or medium-sized crepitations are heard. These crepitations are very characteristic. They occupy the greater part of the inspiratory murmur, although they may also be heard during expiration. They are most frequently heard below the clavicle, and are almost invariably associated with tubercle bacilli in the sputum. Above the clavicle a single sharp clicking crepitation is not infrequently heard at the end of inspiration. When the disease originates in the peribronchial tissue, rhonchi and sibilations are frequently heard, but their significance depends upon their being heard over a restricted area. Pleuritic rub is frequently an indication of early tuberculous infection. It may be due to the presence of tubercles in the pleura, or arise from inflammatory changes following the development of tubercle in the sub-pleural lymphatic tissue. Friction sound heard over the upper lobes is especially significant. The alteration in the character of the breath-sounds heard in early cases of pulmonary tuberculosis is varied. There may be (*a*) alteration in pitch; (*b*) alteration in intensity; (*c*) alteration in duration; (*d*) lengthening of pause between inspiration and expiration; and (*e*) breaking up of the breath-sounds. In his clinical studies it is important that the student should be familiar with the character of the normal breath-sounds before listening to the alteration due to morbid changes in the lung. Feeble or absent breath-sounds over a restricted area is not infrequently due to tuberculous disease of the lung. Irregular breathing, in which the respiratory murmur is broken up, is significant when heard over one lobe or part of a lobe; irregularity of expiration is more significant than that of inspiration. Accentuated breathing is a frequent sign of early apical disease. Inspiration is frequently raised in pitch, and this is one of the earliest indications of pulmonary tuberculosis; expiration also becomes prolonged and harsh, while the interval between both elements is lengthened.

### The Grouping of Certain Diagnostic Indications.

The diagnosis of early pulmonary tuberculosis should not be based on one sign or symptom alone. In the absence of a definite confirmatory test it must be the logical conclusion of the grouping of certain diagnostic indications. In young adults languor, morning cough, and a sub-normal temperature constitute a frequent early combination. Dyspeptic symptoms associated with anæmia and night sweating should always lead to examination of the chest. The dyspepsia of early pulmonary tuberculosis is not infrequently associated with morning sickness induced by the hawking of scanty, tenacious sputum. Muscular wasting associated with persistent headache and neurasthenic symptoms occurring in a young adult may be traced in the majority of cases to certain toxins of the tubercle bacillus which have a selective affinity for the neuro-muscular system. Breathlessness, rise in temperature, and loss of strength are met with in pulmonary tuberculosis of acute onset. In children the diagnosis of latent pulmonary tuberculosis is extremely difficult. Opinions differ as to what combination of clinical data should constitute a trustworthy basis for diagnosis. The diagnosis may usually be based on the association of physical signs or persistent localising symptoms with one or more of the following: (1) certain stigmata of tuberculous infection; (2) positive von-Pirquet reaction; (3) persistent evening pyrexia.

### Confirming the Diagnosis.

In all cases of suspected early pulmonary tuberculosis an effort should be made to confirm the diagnosis. The first step to take is to have the sputum examined, if such be present, for tubercle bacilli. In many cases of what appears

(*Concluded on page* 150.)

### The Early Diagnosis of Pulmonary Tuberculosis.

(*Continued from page* 148.)

clinically to be pulmonary tuberculosis, tubercle bacilli are not found in the sputum. In a certain proportion of these negative cases inoculation of guinea-pigs with the sputum is followed by tuberculosis. In a further proportion of these cases the pneumo-coccus or the pneumo-bacillus will be found to be the causative agent. In the absence of tubercle bacilli in the sputum the diagnosis of pulmonary tuberculin may be confirmed by the induction of a focal reaction with tuberculin. The temperature should be carefully taken for ten or fourteen days, and then .005 c.c. O.T. may be inoculated; if this fail to induce a focal reaction .01 c.c. O.T. may be given. A focal reaction is indicated by chest pains, increased cough and expectoration, and occasionally a streaky hæmoptysis; at the same time physical signs may become more marked, while there is a general reaction with rise in temperature. It is not, as a rule, advisable to inoculate children with O.T., and it is not necessary to increase the dose beyond .01 c.c. Inoculations of O.T. should never be given if slight variations of temperature are present. The presence of pulmonary tuberculosis may be confirmed by *x*-ray examination. Deficient movement of the diaphragm, failure of one apex to clear up on expansion, and especially mottling radiating from the roots, are signs of early tuberculous invasion. There are other tests which indicate the presence of tuberculous infection but do not locate the seat of the disease. Calmette's ophthalmic reaction, Moro's inunction reaction, and von Pirquet's cuti reaction indicate a past or present tuberculous infection, but they are only of assistance in the diagnosis of pulmonary tuberculosis in combination with certain signs and symptoms. The swing of the opsonic index has been employed for purposes of diagnosis, and has proved of value; but the possible margin of error is too wide, and the labour of the several observations necessary too great to make it a trustworthy and easy method of procedure.

Recently attention has been paid to the serum diagnosis of tuberculosis, and evidence points to the possibility that in the serum reaction there is provided a specific test for active cases of early pulmonary tuberculosis, as it is usually absent in arrested or healed tuberculosis and in non-pulmonary tuberculosis. In general practice, however, there is no royal road to the early diagnosis of pulmonary tuberculosis. Reliance must still in large measure be placed on careful examination of the patient, observation of temperature variations, and examination of the sputum.

